# Integral Equation Theory for Coarse-Grained Modeling of Protein Hydration

**DOI:** 10.3390/biom16091242

**Published:** 2026-08-27

**Authors:** Gennady N. Chuev, Timur V. Mamedov, Dmitry O. Morozov

**Affiliations:** Institute of Theoretical and Experimental Biophysics, The Russian Academy of Sciences, Institutskaya St., 142290 Pushchino, Moscow Region, Russia; attore7777777@gmail.com (T.V.M.); dogherty@list.ru (D.O.M.)

**Keywords:** integral equation theory, hydration structure, coarse-grained modeling, SPICA force field, bridge functions, amino acids, proteins

## Abstract

Hydration plays an essential role in protein–protein interactions. Coarse-grained modeling provides an efficient way to treat hydrated protein complexes without the use of extra-large computational resources. To enhance the capabilities of coarse-grained modeling, we developed an integral equation theory based on the solution of the Ornstein–Zerinke equation to evaluate the hydration structure of peptides and proteins within the framework of coarse-grained modeling. Our current version is based on the SPICA force field, which considers distance-dependent interaction potentials between solvent particles and amino acid segments. Our approach involves two key procedures: an accurate estimation of the structure factor of the uniform fluid and the specific construction of bridge functions obtained from MD simulations. The use of a special hybrid closure allows us to reproduce not only the details of the structure factor, but also the isothermal compressibility obtained from the simulations. The developed bridge functions include two components: an analytical repulsive contribution, which is primarily responsible for the thermodynamic properties, and an attractive contribution obtained from MD simulations. The main assumption in the construction is that the contribution of individual amino acids to the attractive bridge function is additive. By parameterizing the bridge functions, we reproduced details of the hydration structure and accurately calculated the hydration energy for various peptides and proteins. Our method is computationally inexpensive and appears to be suitable for the rapid processing of hydrated proteins of any size.

## 1. Introduction

Hydration plays an essential and sometimes crucial role in the functionality of peptides and proteins (see, for example, [1]). The phenomenon has been the focus of numerous studies during the last six decades (see, for instance, [2,3,4]). Understanding hydration at a deep level remains a challenge, despite the vast amount of accumulated experimental data. The reason for this seems to be an inherent synergy that results from a delicate balance between forces involved in the hydration process. Molecular modeling is one of the most powerful methods that enables one to reveal various molecular details. It allows us to properly treat multi-scale interactions occurring during the process and quantify changes in protein structure due to hydration. All-atom molecular dynamics (MD) has become one of the most popular methods for investigating hydrated peptides and proteins. The method provides a deep understanding of the phenomenon, yielding detailed information on the thermodynamic behavior and structural changes in hydrated biomolecules (see, for example, [5,6,7]).

Despite great progress in atomistic MD studies, the currently available computer resources limit the applicability of all-atom MD simulations to biomolecular complexes whose sizes do not exceed nanometers. To overcome this obstacle, coarse-grained (CG) models have been developed. Within the framework of CG treatment, groups of several atoms are presented as a single bead, while interactions between the CG beads are modeled by effective potentials. This involves enthalpic and entropic contributions obtained by averaging certain molecular details. Coarse-grained modeling greatly enhances the power of MD simulations and significantly extends the approach to spatial and temporal ranges that are unavailable for all-atom molecular dynamics. There are numerous brilliant examples demonstrating the effectiveness of CG modeling (see, for instance, [8,9,10]). The approach can be seen as a versatile platform for investigating hydration in various biophysical applications. However, CG molecular dynamics is still limited by the available computational power. The high computational costs of the sampling procedure appear to be another bottleneck for the widespread application of CG MD studies. Therefore, a computational framework is needed to efficiently handle hydration while still preserving the key aspects of CG modeling.

The integral equations theory (IET) can provide an effective solution to this issue [11]. The IET offers many advantages, with the most important of them being: (i) enabling the treatment of an infinite number of solvent particles (without the need for a simulation box) and (ii) simultaneous evaluations of the structural and thermodynamic properties of the solvent and solutes. From a practical standpoint, the main merit of the IET is that, starting with the same input as MD, it can provide the same relevant output at a computational cost of two or three orders of magnitude smaller. In general, the IET method should be considered as a complement to MD studies, offering a way to explore systems that are still limited by computational costs in MD simulations.

The IET approach has been widely used for simple fluids [12,13]; however, there is a single IET application for the CG MARTINI model [14]. The latter model [15] assumes the conventional Lennard-Jones form of interaction potentials between CG particles. Therefore, the application of the IET is straightforward for the model. The goal of the paper is to extend the IET approach to other CG models that involve more complicated nonbonded potentials. The intrigue of this application stems from the inherent dichotomy of CG models. The CG approach simplifies molecular models and treats water blobs with complex structures as CG beads. In this sense, the IET application is similar to that in simple fluids. The CG interaction potentials are much more complicated than those for simple fluids due to an implicit treatment of hidden molecular details. As a result, the CG model can exhibit unexpected results in the case of IET applications.

There is a wide variety of water CG models available. In this study, we focused on the SPICA force field (FF) [16]. Our choice was motivated by the fact that the CG model seems to have a reasonable balance between structural simplification and the inclusion of molecular details necessary to reproduce the thermodynamic and structural properties of hydrated proteins. SPICA FF was originally developed as the Shinoda–DeVane–Klein model [16] for the fast computing of lipid membranes and surfactants. It has since been expanded to incorporate interactions with proteins, nanoparticles, and nuclear acids through systematic CG parameterization [17,18].

The scope of our paper is the following. First, we outline the conventional IET method and demonstrate how it can be extended to CG models (Section 2). We focused on the construction of special bridge functions that allowed us to evaluate the thermodynamic and structural characteristics of protein hydration. Details of the MD simulations and IET calculations are given in Section 2. These bridge functions were derived and parameterized from the SPICA simulations of hydrated amino acids (Section 3.2). In the final stage, we validated and tested the developed approach by calculating the hydration structure around various oligopeptides and proteins (Section 3.3). Finally, we briefly discuss the results obtained and the possibility of extending the approach to other systems.

## 2. Materials and Methods

### 2.1. Integral Equation Theory

As mentioned earlier, the SPICA model considers water to be a fluid consisting of CG beads. Then, to calculate the thermodynamic and structural properties of hydrated solutes within the IET framework, we have to calculate the three-dimensional (3D) solvent density ρ(R). The latter is given by the 3D distribution function $g\left(R\right)=\rho \left(R\right)/\rho_{0}$, where $\rho_{0}$ is the average solvent density of the corresponding uniform fluid. On the other hand, the 3D distribution function can be expressed in terms of the mean-force potential W(R) as [19]:

(1) $$g\left(R\right)\equiv \exp\left[-\beta W\left(R\right)\right]=\exp[-\beta U\left(R\right)+\rho_{0}\int C_{2}(|R-R_{1}|)h\left(R_{1}\right){\mathrm{dR}}_{1}+B\left(R\right)]$$

where $\beta ={\left(k_{B}T\right)}^{-1}$ is related to the temperature *T*, while U(R) is the solute–solvent interaction potential, h(R)=g(R)−1, and B(R) is the bridge function, which takes nonlinear contributions to the mean-force potential into account. In the above equation, the function $C_{2}\left(R\right)$ is the second-order direct correlation function of the uniform fluid. Its Fourier transform (FT) is given by the Fourier-transformed structure factor $S_{0}\left(k\right)$:

(2) $$C_{2}\left(k\right)=1-S_{0}^{-1}\left(k\right)$$

This structure factor is related to the isothermal compressibility $\kappa_{T}$ and can be written in terms of the radial distribution function (RDF) $g_{0}\left(R\right)$ of the uniform fluid:

(3) $$\begin{array}{ccc} & S_{0}\left(k\right) & =1+\rho_{0}h_{0}\left(k\right)\;S_{0}\left(k=0\right)=\rho_{0}k_{B}T\kappa_{T},\\ & h_{0}\left(k\right) & =\int \left[g_{0}\left(r\right)-1\right]e^{-i\mathrm{kR}}\mathrm{dR}=4\pi \int \left[g_{0}\left(r\right)-1\right]\frac{\sin(kr)}{kr}r^{2}dr \end{array}$$

We can redefine the integral contribution to (1) in terms of the direct correlation function c(R) with the use of (2):

(4) $$h\left(R\right)-c\left(R\right)=\rho_{0}\int C_{2}(|R-R_{1}\left|\right)h\left(R_{1}\right){\mathrm{dR}}_{1}$$

which may be treated as an implicit definition of c(R) and rewritten as the conventional Ornstein–Zernike equation:

(5) $$h\left(R\right)=c\left(R\right)+\rho_{0}\int h_{0}(|R-R_{1}\left|\right)c\left(R_{1}\right){\mathrm{dR}}_{1}$$

Therefore, our initial statement is quite similar to that in MD. We used $\rho_{0}$, *T*, and U(R) as the input data, as in the molecular simulations. Then, to evaluate $S_{0}\left(k\right)$ and choose the approximation for B(R), we must solve the set ((1), (5)) for calculating the solvent density ρ(R) and all other required thermodynamic values. Details of the evaluations of the structure factor $S_{0}\left(k\right)$ and bridge functions are provided in the next sections. At this level of consideration, the IET application is similar to that in simple fluids, demonstrating complexity only in the calculations of the 3D distribution functions.

### 2.2. Evaluations of Bridge Functions

Although Formula (1) is quite general, an exact expression of B(R) is not known. Historically, various analytical approximations of B(R) have been used, treating it as a functional of h(R), c(R) or γ(R)=h(R)−c(R). Such approximate closures are commonly derived through a diagrammatic analysis [11,12]. In certain cases, information on the behavior of bridge functions outside the repulsive core can be obtained by MD simulations [20,21,22]. We combined both approaches and express the bridge function as

(6) $$B\left(R\right)=B_{a}\left(\gamma \left(R\right)\right)+B_{md}\left(R\right)$$

where $B_{a}\left(\gamma \left(R\right)\right)$ is an analytical contribution for thermodynamic consistency, while $B_{md}\left(R\right)$ is its counterpart derived from MD simulations and used by us to enhance the quality of the calculated solvent density outside the repulsive core.

Regarding the analytical part of the bridge function, we tested several approximations (see Section 3) and identified the most suitable to be the modified Verlet (VM) closure [23]:

(7) $$B_{a}\left(\gamma \right)=-\frac{A\gamma^{2}}{1+C\gamma }$$

where *A* and *C* are parameters that can be fitted to improve the thermodynamic consistency. We optimized the coefficients to accurately reproduce the experimental hydration energy of amino acid side-chain analogs (see details below).

The bridge function $B_{md}\left(R\right)$ is a complex 3D function in most cases. Moreover, it is subject to high statistical noise and is not well suited for analytical treatment. In order to establish a working protocol, we assumed $B_{md}\left(R\right)$ to be presented as superposed contributions of individual solute beads:

(8) $$B_{md}\left(R\right)=\sum_{i}b_{i}(|R-R_{i}\left|\right)$$

The bridge functions $b_{i}\left(R\right)$ are only distant-dependent and can be derived using the relevant MD RDF $g_{i}\left(R\right)$ as

(9) $$b_{i}\left(R\right)=\ln\left[g_{i}\left(R\right)\right]+\beta U\left(R\right)-\gamma_{i}\left(R\right)-B_{a}\left(\gamma_{i}\left(R\right)\right)$$

where $\gamma_{i}\left(R\right)$ is the indirect correlation function obtained via the OZE solution (5). We note that this function implicitly depends on $b_{i}\left(R\right)$, and hence, relation (9) is to be solved self-consistently. Then, the derived bridge functions can be parameterized. We applied the analytical formula:

(10) $$b_{i}\left(R\right)=p_{1}\left(R\right)\epsilon_{i}\left[{\left(\frac{\sigma_{i}}{R}\right)}^{28}-{\left(\frac{\sigma_{i}}{R}\right)}^{4}\right]+p_{2}\left(r\right)\xi_{i}\cos\left(\pi R\right)$$

where $\sigma_{i}$, $\epsilon_{i}$ and $\xi_{i}$ are parameters that depend on the choice of the CG bead, while $p_{1}\left(r\right)$ and $p_{2}\left(r\right)$ are switching functions (see details in the Supplementary Information). We used a high power (specifically, 28) to achieve a fast decrease at short distances and added a cosine function to account for oscillations at longer distances. Thus, by utilizing only these three parameters and applying (10), we can calculate $B_{md}\left(R\right)$.

### 2.3. Evaluations of the Structure Factor and Isothermal Compressibility

There are various approaches for evaluating the structure factor of a uniform fluid by extension of the RDFs obtained from molecular dynamics [24,25,26]. However, it is not guaranteed that this method will accurately reproduce the isothermal compressibility related to the value $S_{0}\left(k=0\right)$ in this way. In order to provide both the RDF and the isothermal compressibility obtained by molecular dynamics, we combined the OZE

(11) $$h_{0}\left(r\right)=c\left(r\right)+\rho_{0}\int h_{0}(|r-r_{1}\left|\right)c\left(r_{1}\right){\mathrm{dr}}_{1}$$

with a hybrid closure relation. For this purpose, we utilized molecular dynamics to evaluate $g_{md}\left(r\right)=h_{md}\left(r\right)+1$ at relatively short distances $r<r_{md}$. Then, we extended the distance range by applying the modified hybrid closure

(12) $$h_{0}\left(r\right)=h_{IET}\left(r\right)+\left(1-\alpha \right)p\left(r\right)\left[h_{md}\left(r\right)-h_{IET}\left(r\right)\right]$$

where $h_{IET}\left(r\right)$ is calculated by the KH closure (13) and α<<1 is a small factor, while p(r) is a smooth switching function, i.e., $p(r<r_{1})=1$ and $p(r>r_{md})=0$. Due to this modification, the RDF is insignificantly different from MD data at short distances: $g\left(r\leq r_{1}\right)=\alpha g_{IET}\left(r\right)+\left(1-\alpha \right)g_{md}\left(r\right)\approx g_{md}\left(r\right)$. On the other hand, we have $g\left(r>r_{md}\right)=g_{IET}\left(r\right)$. By fitting the coefficients for the VM bridge function, we can easily reproduce the isothermal compressibility obtained within the molecular dynamics. The parameters $\alpha ,r_{1},r_{MD}$, as well as the expression of p(r), are given in the Supplementary Information.

### 2.4. Details of Computations

We used the SPICA CG model for water, peptides, and proteins. In the SPICA model [16,18], a single CG water bead contains three water molecules. The FF parameters for the SPICA water model are the same as those previously published [16]. Regarding peptides, SPICA FF treats backbone groups as a single CG particle, while side-chain groups are considered to be segments containing 0 to 4 CG beads depending on the specific amino acid [18]. An example of a typical CG representation of tetra-valine can be observed in the Supplementary Information (Figure S1). Details of the CG mapping of the 20 amino acids in the SPICA FF are given in the original paper, as well as information on the FF parameters [18]. Initially, the protein structures were obtained from the protein data bank [27] (see schematic view in Supplementary Information). The CG models were derived from the all-atom structures using the SPICA FF. The procedure followed a topology-based conversion protocol. We generated initial configurations and prepared CG topology files using the SPICA website (https://www.spica-ff.org/). CG amino acids and proteins were positioned at the origin of the cubic simulation box, which had a size of 40 Å for amino acids and was extended to 70 and 90 Å for simulations of proteins such as insulin and protein dimers.

We performed MD simulations using the LAMMPS software package 2024 [28]. The calculations were conducted in three stages: energy minimization of the system, equilibration, and MD simulation runs. The energy minimization was carried out until an energy gradient of $10^{-6}$ kcal/mol was reached. The equilibration was conducted in the NVT ensemble at T = 298.15 K; the stage involved $10^{4}$ steps with a time step of 1 fs. The main MD runs were conducted in the NVT ensemble at a temperature of 300 K and a density of CG water particles equal to 0.01109 Å^−3^, which corresponded to the normal conditions for water. The isothermal compressibility $\kappa_{T}$ for bulk water was evaluated in the NPT ensemble by calculations of volume fluctuations, i.e., $\kappa_{T}=(\langle V^{2}\rangle -{\langle V\rangle }^{2}({\left[k_{B}T\langle V\rangle \right]}^{-1}$. The total simulation run involved 2 ×$10^{6}$ steps with a time step of 10 fs. We saved the trajectories every 200 or 500 steps, recording the atom IDs, the molecule numbers, the atom types, and their coordinates. The MDanalysis package [29] was used to analyze the trajectories and construct various 1D and 3D correlation functions. The density data were recorded with a spatial resolution of 0.5 Å, while the bin size was equal to 0.1 Å for the RDF calculations.

### 2.5. IET Calculations

To improve the robustness of the developed algorithm, we made a few modifications to the conventional IET method. First, we limited our applications of the VM bridge functional to the range where γ(r)>0 to prevent a divergence of the denominator in Equation (7). Second, we partially linearized the exponent in Equation (1) to enhance the convergence of the solution, similar to the method used in the Kovalenko–Hirata (KH) [30] approximation, i.e.,

(13) g(R)=E(−βU(R)+h(R)−c(R)+B(R))−1

where function E(a) is defined as E(a<0)=exp(a),E(a>0)=1+a. This modification not only improves the convergence of the solution, but also substantially decreases the difference between the simulated and calculated distribution functions. The latter simplifies their analytical treatment.

The number of grid points was $2^{7}$ with a grid step of 0.05 Å for the 1D evaluations of the uniform SPICA water. In the 3D IET study, we used the calculation boxes with sizes equal to those used in the MD simulations with a grid step of 0.5 Å and a tolerance of $10^{-6}$ for all calculations. To accelerate the IET iterations for both 1D and 3D calculations, we applied the MDIIS solver [31].

The same thermodynamic conditions and SPICA FF parameters for nonbonded CG interactions were used as in the MD simulations. The hydration free energy was calculated using an analytical formula:

(14) $$\begin{array}{ccc} & \Delta G & =\Delta G_{KH}+\Delta G_{B}\; \end{array}$$

where $\Delta G_{KH}$ is an expression for the free energy within the KH approximation [30], while $\Delta G_{B}$ is the contribution due to the nonzero bridge functional. The analytical expressions for $\Delta G_{KH}$ and $\Delta G_{B}$ in terms of h(R) and c(R) are given in the Supplementary Information.

## 3. Results

### 3.1. Uniform SPICA Water

**Figure 1:**
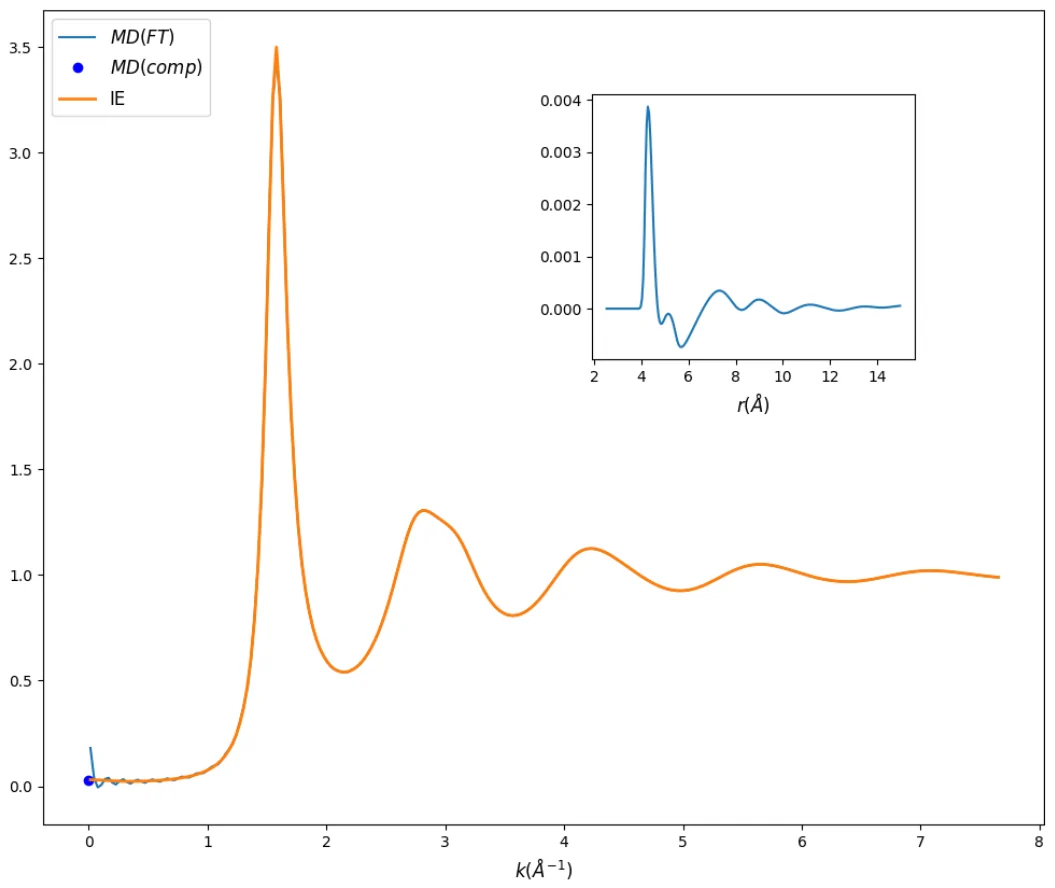
Structure factor of uniform SPICA water obtained by the FT of the MD RDF (blue curve) and by the IET (orange curve). The blue circle indicates the value of S(k=0) derived from volume fluctuations. The inset demonstrates a difference between the RDFs calculated by the IET and MD methods.

First, we applied the method to obtain the structure factor of the uniform SPICA water under normal conditions. To do this, we extended the RDF derived from the molecular simulations. We note that the structure factor obtained directly by the Fourier transform of the MD RDF revealed strong oscillations at low wave vectors (see Figure 1) and was not appropriate for the IET calculations. The effect appears to be caused by finite-size effects [32]. Although there is a sophisticated technique available to suppress oscillations [33], it does not allow for proper calculations of the isothermal compressibility. To overcome this bottleneck, we evaluated the volume fluctuations by MD. Then, we carried out the IET calculations by varying coefficients *A* and *C* in the VM bridge functional. Finally, we fit the coefficients to match the value of $S_{0}\left(k=0\right)$, calculated by the IET with the MD data. Figure 1 demonstrates the structure factor of uniform SPICA water obtained by MD and the IET. As can be seen, the IET reproduced, with high precision, the MD structure factor at enough large wave vectors k>0.5 Å^−1^. On the other hand, the MD evaluations of $S_{0}\left(k=0\right)$ were not suitable at lower wave vectors. Nevertheless, our IET protocol offers accurate calculations of the structure factor and yields the same isothermal compressibility as that derived from volume fluctuations. At the same time, the difference between the RDFs calculated by MD and the IET was minimal, less than the statistical noise, and can be negligible (see the inset in Figure 1). We tested the impact of various numerical parameters of our combined closure by varying them across a wide range. We determined that the changes in the parameters do not affect the results, but lead only to variations in the fitted coefficients of the VM bridge functional. Detailed information on the coefficients and parameters of the switching function is given in the Supplementary Information.

Therefore, the IET treatment of uniform SPICA water is robust and accurate enough to evaluate the main thermodynamic and structural characteristics of the water. Our comparative analysis indicated that the IET is quite able to predict the static structure factor and the RDF. However, the application of the hybrid closure allows us to reproduce the isothermal compressibility with a high accuracy.

### 3.2. Hydrated Amino Acids

Initially, we evaluated RDFs for various side-chain segments within the framework of the IET approach, using the obtained structure factor and various closures. Figure 1 shows the results of such calculations and compares them with MD data obtained for the THR particle. As can be seen, the hypernettetd chain (HNC) approximation (B≡0) strongly overestimated the distribution function, whereas the KH closure underestimated the RDF. Similar results have been obtained for other side-chain segments (see Figure S2 in the Supporting Information).

**Figure 2:**
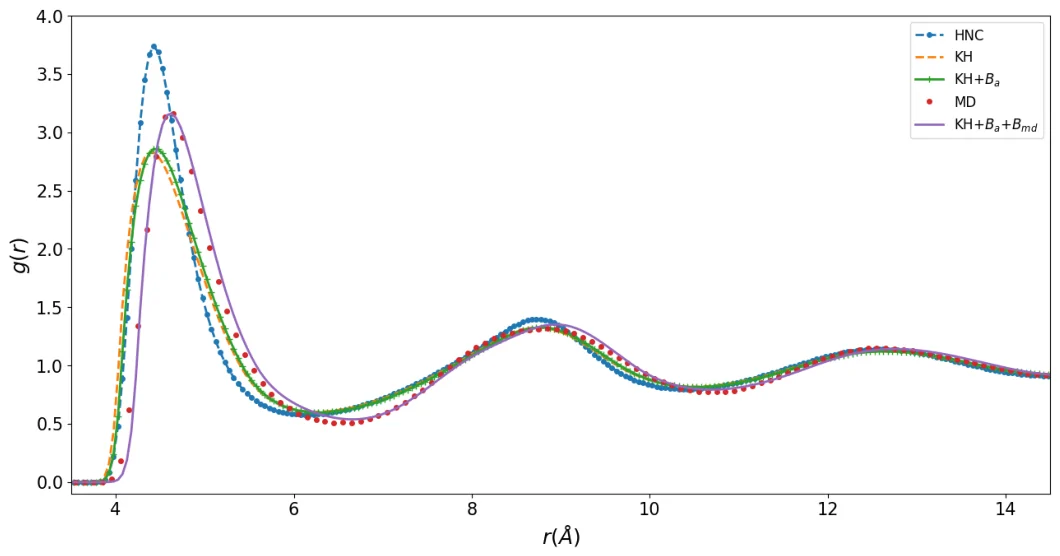
RDF of hydrated THR segment calculated by the MD (dots) and IET with the $HNC,\;KH,\;KH+B_{a}$ and $KH+B_{a}+B_{u}$ closures.

As expected, the application of SPICA FF led to RDFs with a more complicated behavior than those for CG Lennard-Jones fluids [14]. Neither the HNC nor the KH closures provided satisfactory evaluations of RDFs. To improve the performance of the IET calculations, we applied the VM bridge functional. We optimized the coefficients *A* and *C* of the VM functional to reproduce the hydration free energies ΔG derived from MD and minimized the deviations from the simulated RDFs. The results obtained for the hydration energy are listed in Table 1 together with the values of the root-mean-squared deviation (RMSD) between the calculated and simulated data. We note that the table does not show the hydration free energies for amino acids that have no analogs (charged amino acids and Gbm bead). The calculated free energies were in good agreement with those obtained by MD; however, the application of the VM bridge functional did not improve the quality of the RDF calculations (see Figure 2). The calculated RMSDs demonstrated a pronounced difference between the simulations and calculations (see Table 1). We not only tested the RDFs for single-site side-chain segments, but also evaluated the 3D solvent density ρ(r) around multi-site amino acids such as Phe, Tyr, and Trp. Table 1 indicates that the calculated RMSD for multi-site amino acids decreases strongly due to a highly increased number *N* of grid points with respect to that in the 1D calculations, since the RMSD $\propto 1/\sqrt{N}$.

**Figure 3:**
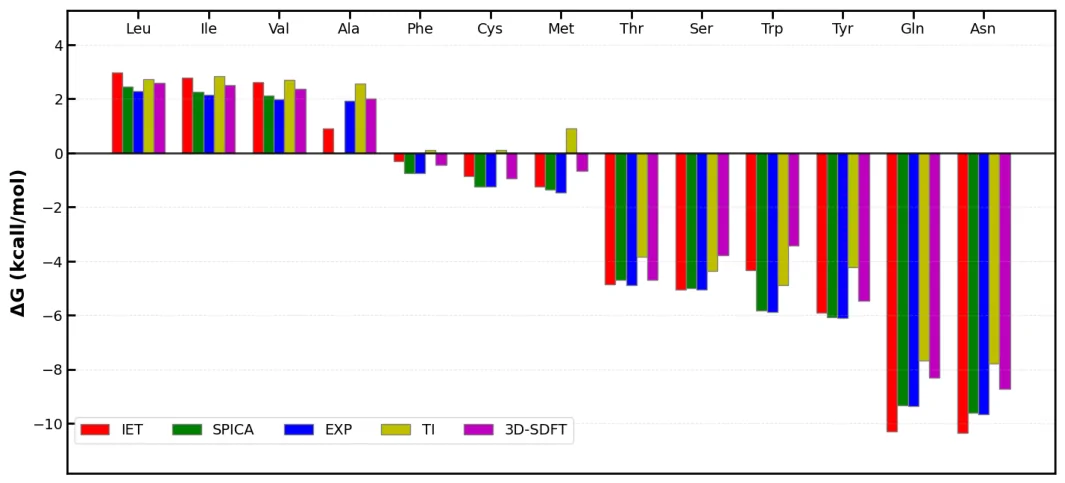
Hydration free energies obtained by the CG MD (green) [18] and CG IET (red), all-atom MD (khaki) [34] and all-atom 3D site-density functional approach (pink) [35] and by the experiment (blue) [34].

To estimate the performance of our approach, we compared the calculated hydration free energies with those obtained by various methods involving atomistic MD [34] or a 3D site-density functional approach [35], as well as the experimental data [36] (Figure 3). All the methods except the CG MD provided more/less similar estimates, whereas the CG MD yielded an extremely high precision. This accuracy can likely be attributed to the CG protocol, specifically a procedure that adjusts the interaction parameters of the individual beads to reproduce the experimental free energies. By applying the individual coefficients $A_{i}$ and $C_{i}$, we can enhance the accuracy of the calculated hydration energies. However, we limited our optimization by only two coefficients that were the same for all the segments. Despite this limitation, when comparing our results to those of other methods, we conclude that the IET yielded reasonable values for hydration free energies, with an accuracy comparable with that obtained using atomistic treatments.

**Figure 4:**
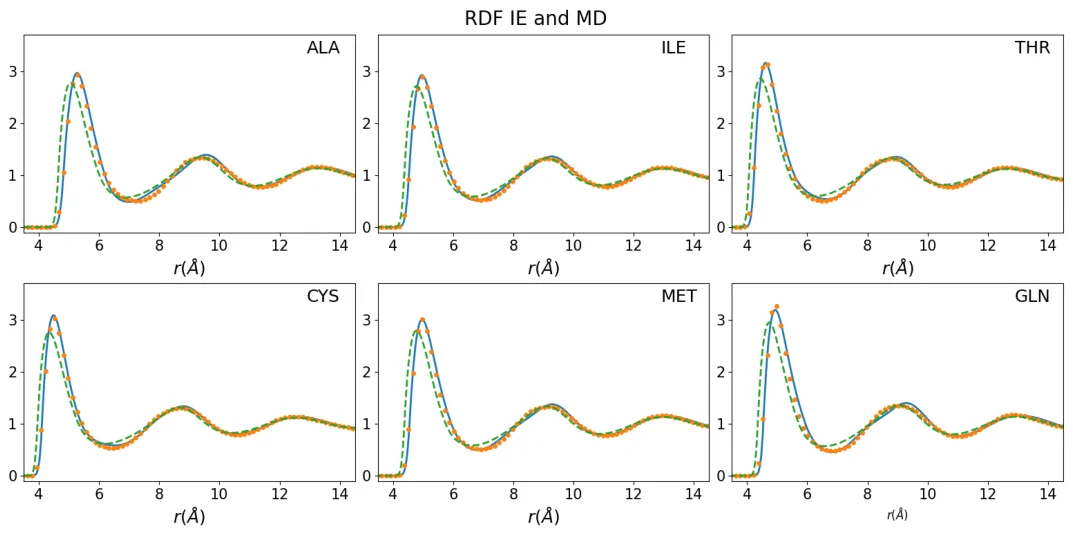
RDFs for various side-chain segments obtained by the MD (dots) and IET with closures $KH+B_{a}$ (dashed curves) and by $KH+B_{a}+B_{u}$ (solid curves).

**Figure 5:**
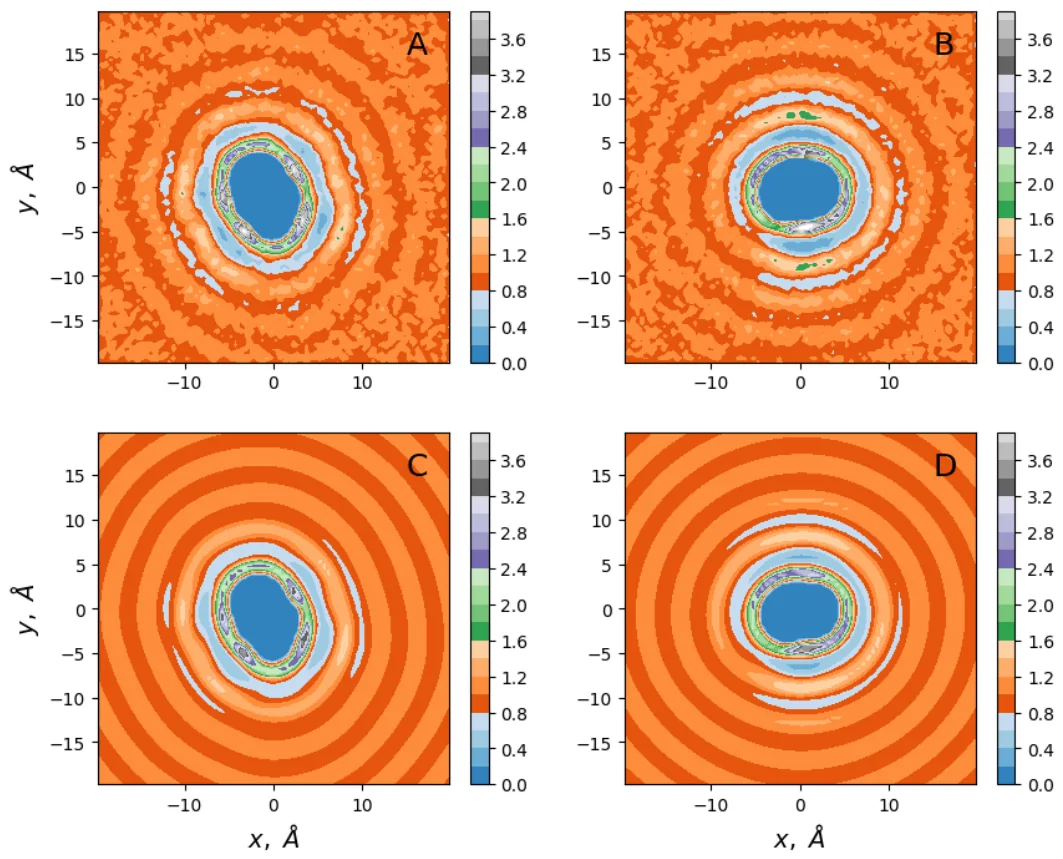
Reduced solvent density $\rho \left(r\right)/\rho_{0}$ for Tyr (**A**,**C**) and Phe (**B**,**D**) calculated by MD (**top**) and IET (**bottom**) with $KH+B_{a}+B_{u}$ closure.

Although the application of the VM bridge functional improved the calculations of the hydration free energy, a discrepancy between the calculated and simulated RDFs remained, especially in the range of the first peaks (Figure 2). Our detailed study revealed that the effect is caused by a nonlinear attraction. The latter is a peculiarity of the CG SPICA model and can be treated as an attractive bridge contribution outside the repulsive core. This phenomenon is unlikely to be revealed in simple fluids, where the bridge function is mainly repulsive. We investigated the attractive bridge function for all the side-chain segments and found that the contribution is rather universal and can be approximated by a simple analytical form (see Section 2.2). We fit the coefficients of this approximation to minimize the RMSD for all the side-chain segments. The results are listed in Table 1, indicating a strong decrease in the RMSD for the improved bridge functions by approximately 5 times (see Table 1). Figure 4 demonstrates the performance of our calculations by comparing the MD and IET data for RDFs calculated by various closures. The use of the derived bridge functions $B_{u}$ allowed us to significantly reduce the discrepancy between the MD and IET data. We also applied the method to evaluate the 3D solvent density around multi-site amino acids. In Figure 5, we compare the reduced solvent density $\rho \left(r\right)/\rho_{0}$ for Tyr and Phe multi-site particles. Although the MD data are a bit noisy, there is clearly an excellent agreement between the MD data and the IET calculations.

Therefore, the developed procedure allowed us to fit and further parameterize the bridge functions derived from MD. By combining the derived bridge functions with analytical bridge functionals, we can provide highly accurate evaluations of the thermodynamic and structural properties of hydrated amino acids. The method can be easily extended to the 3D calculations. We were able to reproduce not only the RDFs, but also the simulated 3D solvent density ρ(r) with a high precision. Our calculations are in good agreement with the CG MD data and experimental measurements of the hydration free energy for amino acid analogs.

### 3.3. Peptides and Proteins

**Figure 6:**
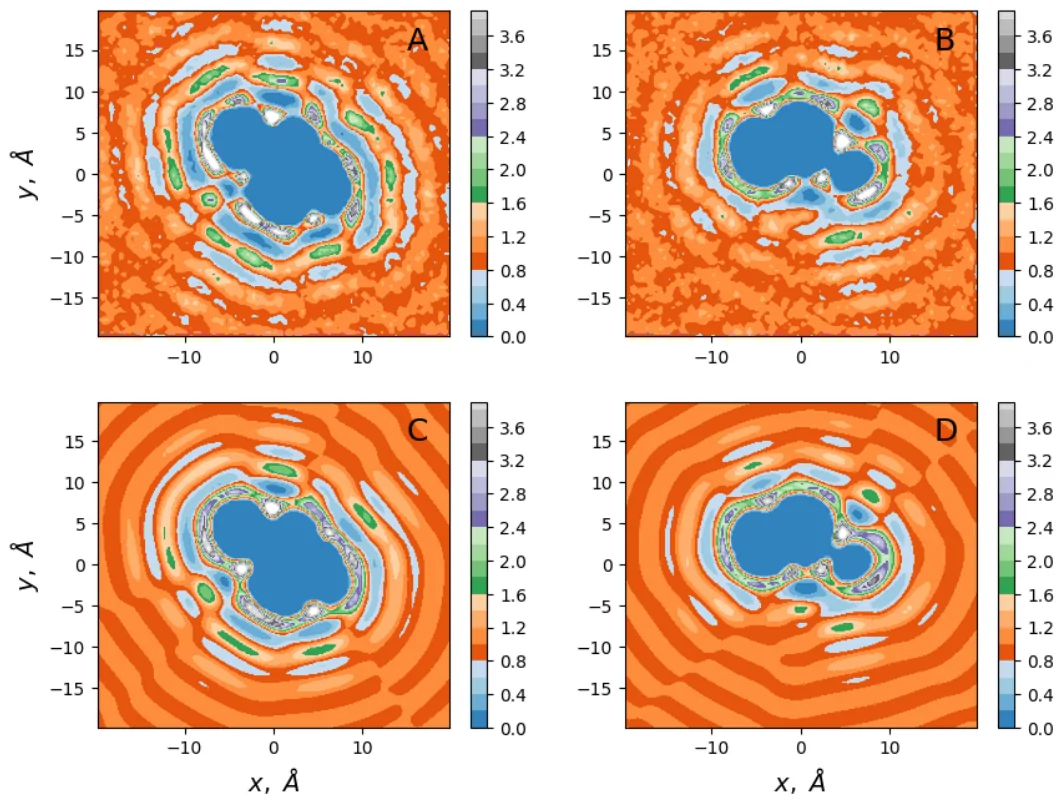
Reduced solvent density $\rho \left(r\right)/\rho_{0}$ around tetrapeptides EEEE (**A**,**C**) and VVVV (**B**,**D**) calculated by MD (**top**) and IET (**bottom**) with $KH+B_{a}+B_{u}$ closure.

In the final step of our investigation, we applied the IET approach together with the sophisticated bridge functions for treating the hydration of oligopeptides and proteins. First, we tested the CG models of tertra-valine and tetra-glutamic acids, which are denoted below as VVVV and EEEE, respectively. The former represents an example of a hydrophobic peptide, while the latter shows an example of a charged solute (the total EEEE charge was equal to 4). We derived structures of the fragments from the relevant PDB data and then ran MD simulations as described in Section 2.4. We used equilibrium configurations of the oligopeptides as the input and then provided the IET calculations. Figure 6 shows the two-dimensional (2D) plan at z=0 of the reduced 3D solvent density $\rho \left(r\right)/\rho_{0}$ around the tetra-peptides. Similar to multi-site side-chain segments, the agreement between the IET calculations and the MD data was excellent. The theory indicates all the peculiarities obtained by MD. Namely, the solvent density around hydrophobic tetra-peptides was more smooth, whereas hydration peaks had a lower amplitude than those for charged peptides. Our calculations support this observation. There is a good quantitative agreement between the MD and IET data, with an RMSD of less than 3 × $10^{-3}$ (see Table 2).

**Figure 7:**
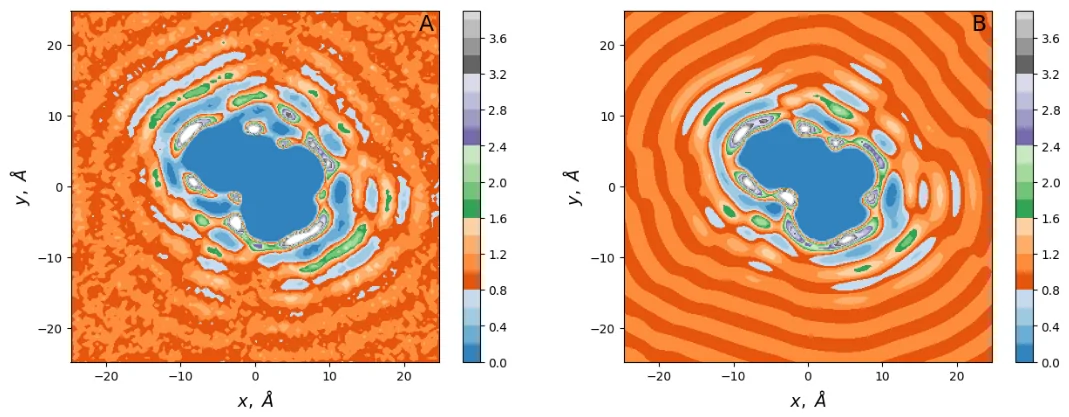
Reduced solvent density $\rho \left(r\right)/\rho_{0}$ around oxytocin calculated by the MD (**A**) and IET (**B**) with $KH+B_{a}+B_{u}$ closure.

Then, we validated the method for oxytocin. This peptide hormone consists of only nine amino acids and has been extensively simulated in various aqueous solutions. Initially, we used the pdb-file (PDB code is 2MGO; see molecular structure in the Supplementary Information), minimized the energy, and then equilibrated the structure. Finally, we performed an MD run and evaluated the hydration structure, calculating the 3D solvent density. The results and their comparison with the relevant IET calculations are shown in Figure 7. Although there was a small discrepancy between the calculated and simulated hydration structures, we observed a reasonable agreement between the MD data and IET calculations.

**Figure 8:**
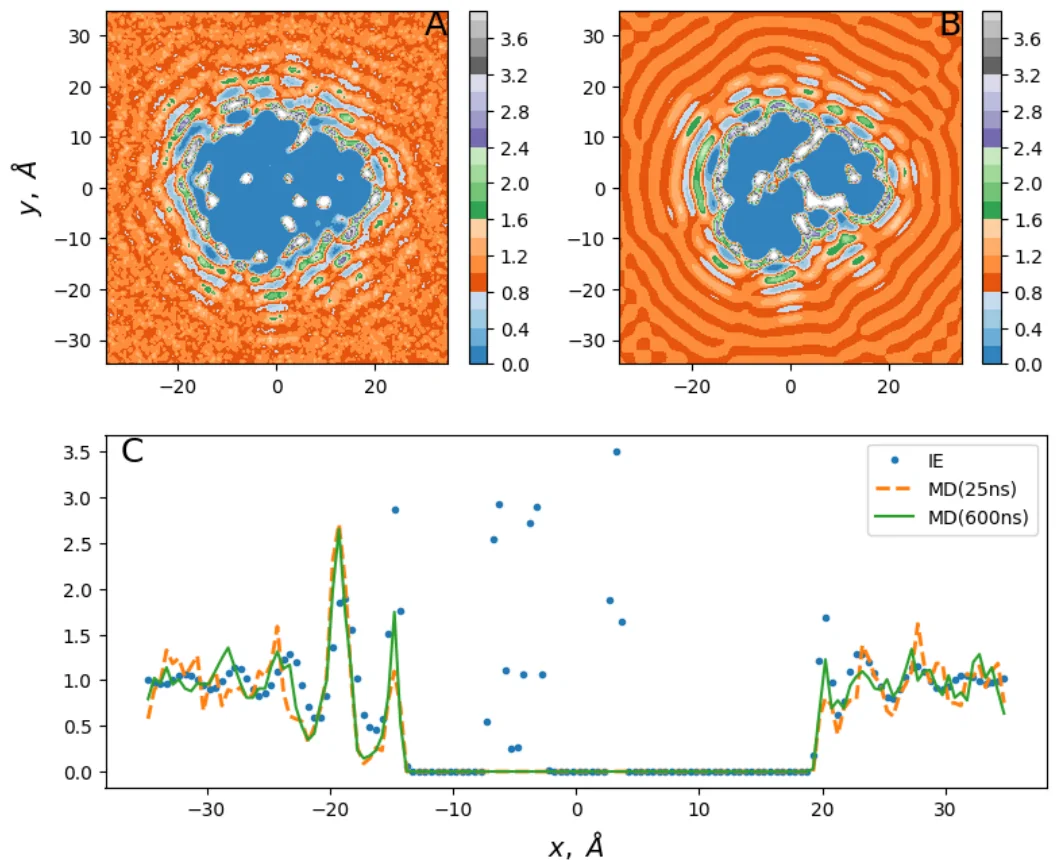
Reduced solvent density $\rho \left(r\right)/\rho_{0}$ around insulin calculated by MD (**A**) and IET (**B**) with $KH+B_{a}+B_{u}$ closure. Part (**C**) shows the 1D water distribution $\rho \left(x,z=0,y=0\right)/\rho_{0}$: IET data indicated by dots, long-time MD by solid curves, short-time MD by dashed curves.

Next, we investigated the hydration of insulin. We used data from the pdb file with code 4F1C as an initial configuration (molecular structure is presented in Figure S3 of the Supplementary Information). The peptide contained 102 residues. The results are demonstrated in Figure 8. As before, the theory and MD simulations were in agreement, especially in the peripheral region around insulin. Small deviations between the data seemed to be caused by statistical noise. We noticed a peculiarity of the insulin hydration. The CG MD study revealed a certain probability of detecting water in the pockets on the insulin surface (see Figure 8). The IET calculations confirmed this effect; however, the theory strongly overestimated this phenomenon. The latter is not surprising, since our parameterization of the bridge functions is only valid outside the protein core and does not allow us to treat water in cavities and nanopores.

**Figure 9:**
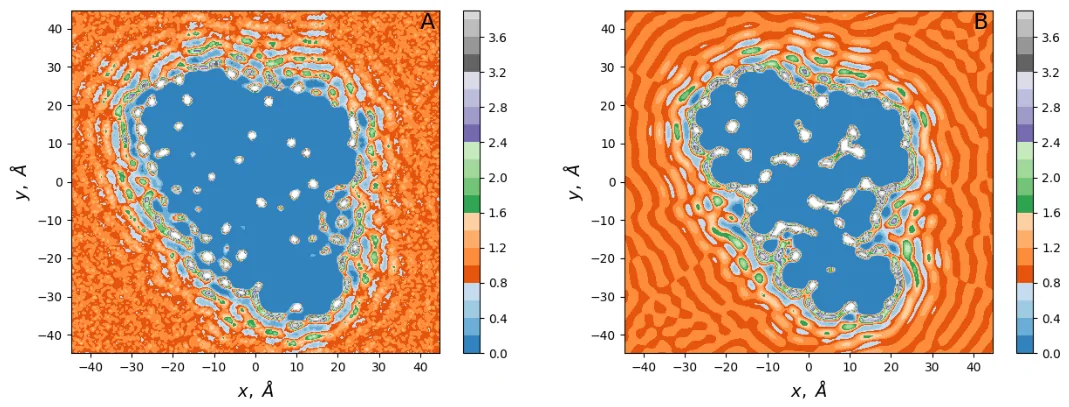
Reduced solvent density $\rho \left(r\right)/\rho_{0}$ around Ras-Raf protein complex calculated by MD (**A**) and IET (**B**) with $KH+B_{a}+B_{u}$ closure.

Finally, we applied the method to investigate the hydration of human H-Ras and the H- Ras-binding domain (RBD) of the C-Raf1 complex. Various structures for this complex have been resolved. We applied the complex structure with the 4G0N pdb code. This structure contains 244 residues and reveals behavior typical of protein–protein complexes. The MD simulations and IET calculations were performed as previously described. The results are depicted in Figure 9. The latter demonstrates the 2D distribution of the solvent density $\rho \left(x,y,z=0\right)/\rho_{0}$ in the (x,y) plane with z=0. As previously observed, the IET data were consistent with the MD simulations for the water species distributed in the peripheral region around the complex. The situation is more complex for internal water sites. Although both methods indicate the presence of water species in cavities inside the complex, the IET overestimated this probability and provided evidence in favor of a more hydrophilic nature of the cavities.

## 4. Discussion

We developed the IET approach to evaluate the hydration structure around peptides and proteins within the framework of CG modeling. The current IET version deals with the SPICA water model treating a water molecule as a single bead. As a result, our calculations of the hydration structure around the proteins were reduced to the solution of the 3D Ornstein–Zernike equation. In general, the approach can be extended to multi-site water models such as pSPICA [37], replacing the OZE with the 3D reference interaction model [24]. Although an extension of the method for polarizable water models is available, it requires the development of a special formalism that treats water flexibility self-consistently.

We focused on applications of the IET to coarse-grained models. An alternative to coarse-grained modeling is multi-scale methods, in particular, hybrid schemes based on combinations of molecular dynamics and hydrodynamics (see, for example, [38,39,40]). Although a combination of the IET with hydrodynamics is possible, such models are unlikely to be effective, since the IET can not treat dynamic effects, and hence, cannot be applied for many problems, which are in focus of the hybrid schemes.

The starting point of our evaluations was an accurate treatment of the structure factor for uniform water. The use of a special hybrid closure for uniform water allowed us to reproduce not only the details of the MD structure factor, but also the isothermal compressibility obtained by the CG MD simulations. Although we modified the KH closure, there are alternatives [26,41]. The key procedure of our method was to construct the suitable bridge functions. The latter involve two counterparts, namely the analytical VM contribution, which is mainly responsible for thermodynamic properties, and the attractive contribution, which is derived from MD simulations. We extracted the bridge functions from the CG MD simulations of the corresponding amino acid segments. We tested various approximations for the bridge functions. In particular, the exponent index for the repulsive term varied in a wide range from 12 to 30, and from 3 to 6 for the attractive part. In general, the larger the index for repulsion, the lower the deviation from MD simulations. Conversely, the smaller the index for attraction, the lower the deviation. However, using an exponent index of 3 for attraction can lead to instability, whereas varying the index for repulsion does not significantly affect the stability of the solution.

We fit parameters to construct reliable bridge functions. A two-parameter bridge function for water was used to reproduce the compressibility of the simulated system. A similar two-parameter repulsive bridge function was used for proteins to reproduce the experimental hydration free energies. To do this, a universal dependence was used for all amino acids. Detailed experimental data on the hydration structure around hydrated proteins are not available. Due to this reason, we optimized the bridge parameters for the attractive part by minimizing the RMSD with respect to the simulated data. By treating the structural properties of the hydration shells, we used individual coefficients for the attractive part of the bridge functions to improve the performance of the model. There are data on the hydration shells around protein–membrane complexes, which were obtained using non-contact atomic force microscopy [42,43]. This issue will be a focus of our future research. To carry out the investigations, we will extend our method to lipids and nanoparticles.

We succeeded in the valid reproduction of the hydration structure outside peptides and proteins, whereas the evaluations of the hydration structure in protein cavities and pockets were less accurate. However, the latter is a common defect in the CG modeling of proteins, since the models are not able to accurately reproduce the geometry of these cavities. Our analysis indicates that the performance of the developed approach decreases slightly as the size of the proteins considered increases. Nevertheless, the method appears to be suitable for the fast treatment of hydrated proteins of various sizes within the framework of CG modeling. In essence, the method can be easily extended to biomolecular complexes with sizes that reach several tens of nanometers. Currently, our studies are limited to evaluations of the hydration structure and free energies. However, it can be easily extended to other thermodynamic properties, such as the mean force potentials or the interaction forces between hydrated proteins. The latter seems to be a promising way to achieve the further extension of the method, and the results will be published elsewhere.

## 5. Conclusions

We developed a method for the fast treatment of the hydration structure around proteins and olygopeptides within the framework of coarse-grained modeling based on the SPICA force field. The water is assumed to be a fluid consisting of CG particles with distant dependent interaction potentials. Proteins and peptides are treated as CG solutes with complex 3D shapes. Our method is based on the solution of the Ornstein–Zernike equation and applies semi-empirical bridge functions. The latter are derived with the use of coarse-grained molecular dynamics and optimized for individual amino acid segments. The method is computationally simple and robust. Typically, only a few minutes of laptop calculations are needed to calculate the 3D solvent density in most cases. Due to the bridge parameterization, our approach allows us to not only evaluate, with high precision, the hydrated structure of various proteins, but also to estimate the hydration free energies of amino acids. Several tests provided by us demonstrated an excellent agreement between the simulated and calculated hydration structures. Although we restricted ourselves to only proteins and peptides, the method can be easily extended to other molecules such as surfactants, as well as lipids and nucleic acids, whose CG modeling is available within the framework of the SPICA force field. The study on the extension of the method to other biomolecules will be published elsewhere.

## Figures and Tables

**Table 1:** Hydration free energies and RMSD between simulated and calculated RDFs for various amino acids.

Amino Acid	ΔG (kcal/mol)		RMSD	
	MD [18]	IET	$\mathrm{KH}+B_{a}$	$\mathrm{KH}+B_{a}$ + $B_{u}$
Val	2.12	2.63	0.168	0.031
Ile	2.26	2.79	0.187	0.036
Leu	2.46	2.97	0.182	0.034
Ser	−5.00	−5.07	0.174	0.059
Thr	−4.70	−4.87	0.188	0.049
Cys	−1.24	−0.87	0.171	0.034
Met	−1.37	−1.25	0.196	0.042
Asn	−9.61	−10.35	0.205	0.064
Gln	−9.34	−10.30	0.215	0.071
Phe	−0.74	−0.32	0.001	0.001
Tyr	−6.08	−5.92	0.001	0.001
Trp	−5.84	−4.35	0.001	0.001
Gbm			0.148	0.046
Asp			0.214	0.077
Ly2			0.206	0.066
Glu			0.212	0.073

**Table 2:** RMSD between 3D-simulated and calculated solvent densities $\rho \left(r\right)/\rho_{0}$ for various oligopeptides and proteins.

Peptide/Protein Name	RMSD
VVVV	0.002
EEEE	0.003
2MGO	0.003
4F1C	0.009
4G0N	0.015

## Data Availability

See the Supplementary Material for switching functions $p_{1}\left(r\right)$ and $p_{2}\left(r\right)$, analytical expressions for free energy contributions $\Delta G_{KH}$ and $\Delta G_{B}$, tables of optimized coefficients for bridge functions (Tables S1 and S2), a typical CG representation of tetra-valine (Figure S1), additional data on the RDFs of amino acid segments (Figure S2), and a schematic view of the molecular structure of the proteins under consideration (Figure S3).

## Supplementary Materials

The following supporting information can be downloaded at: https://www.mdpi.com/article/10.3390/biom16091242/s1, Figure S1: Snapshot of hydrated tetra-valine simulated with the use of the SPICA FF. Backbone atoms are indicated by red, amino acid side-chain segments by green, while water beads are colored by blue; Figure S2: RDFs for various side-chain segments obtained by the MD (dots) and IET with closures *KH* + *B_a_* (dashed curves) and by *KH* + *B_a_* + *B_u_* (solid curves); Figure S3: Molecular structure of oxytocin (a), insulin (b), and Ras-Rasf dimer (c) derived from the pdb database; Table S1: Optimized parameters of bridge function function $B_{a}\left(r\right)$; Table S2: Optimized parameters of bridge function $B_{u}\left(r\right)$ for amino acid segments.
