# Supplementary material for: Integral Equation Theory for Coarse-Grained Modeling of Protein Hydration
Source: Biomolecules. 2026 Aug 27;16(9):1242. doi: 10.3390/biom16091242 (PMC13604487; doi:10.3390/biom16091242)
Supplement: Supplementary file 1 [file biomolecules-16-01242-s001.zip › biomolecules-4503081-supplementary.pdf]

Supplementary Information
Integral Equation Theory for Coarse-Grained Modeling Hydration Structure around Peptides and Proteins

Gennady N. Chuev, Timur V. Mamedov, and Dmitry O. Morozov

Institute of Theoretical and Experimental Biophysics,

Russian Academy of Science, Pushchino, Moscow Region, 142290, Russia

The switching function $p(r)$ is given by

$$
\begin{aligned}
p\ \ (r) &= 1 && r \leq r_1 \\
p\ \ (r) &= \frac{(r - r_{md})^2 (2r - 3r_1 + r_{MD})}{(r_{md} - r_1)^3} && r_1 < r < r_{md} \\
p\ \ (r) &= 0 && r \geq r_{md}
\end{aligned}
\qquad \text{(S.1)}
$$

For our calculations $\alpha = 3 \cdot 10^{-3}$, $r_1 = 22$Å, and $r_{md} = 40$Å.

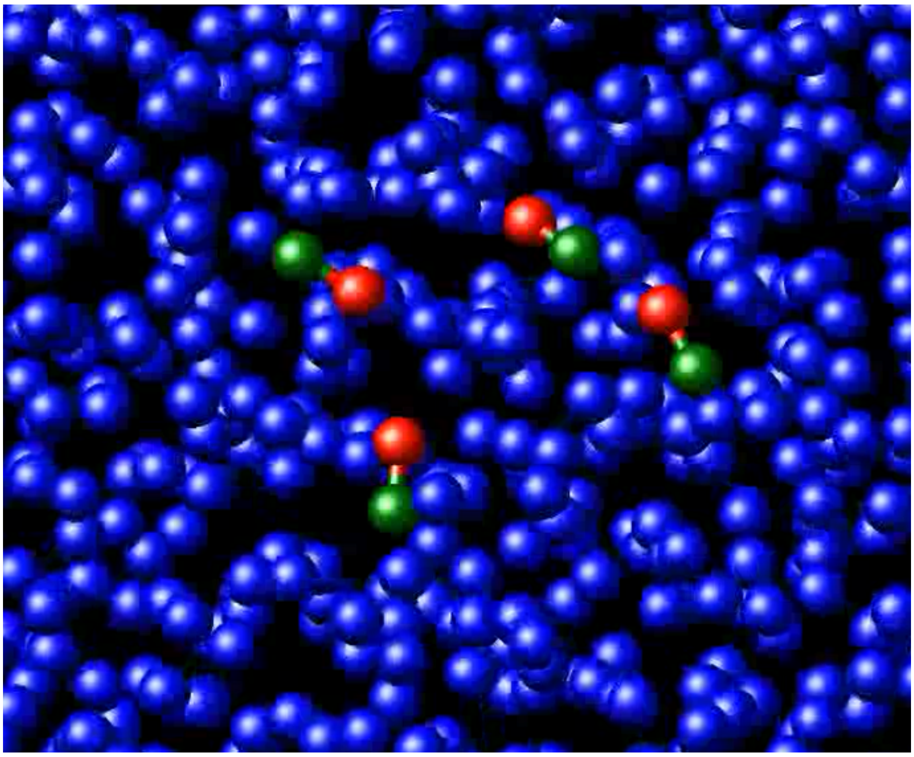

FIG. S.1: Snapshot of hydrated tetra-valine simulated with the use of the SPICA FF. Backbone atoms are indicated by red, amino acid side-chain segments by green, while water beads are colored by blue.

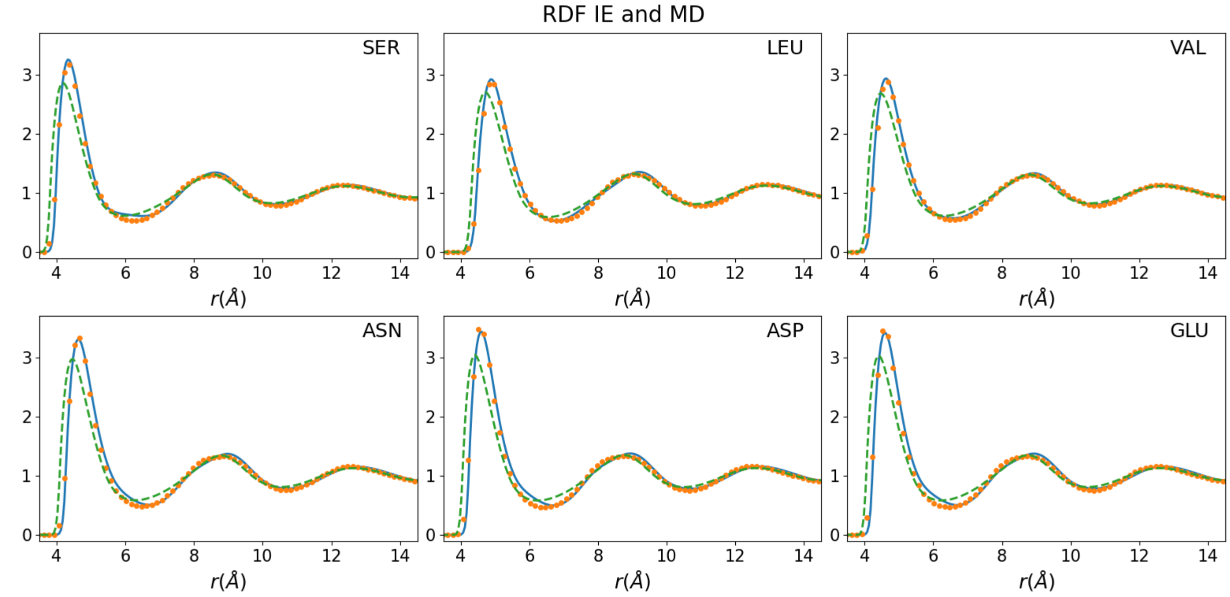

FIG. S.2: RDFs for various side-chain segments obtained by the MD (dots) and IET with closures $KH + B_a$ (dashed curves) and by $KH + B_a + B_u$ (solid curves).

TABLE S.1: Optimized parameters of bridge function $B_a(r)$.

System	A	C
uniform water	0.232	0.3
nonuniform water	0.085	0.071

The switching functions $p_1(r)$ and $p_2(r)$ are expressed as

$$p_1(r) = [1 + \exp 5(r - \sigma_i)]^{-1}$$
$$p_2(r) = p_1(r) f(r)$$
$$f(r) = 0.5 - 0.5 \tanh(0.6r - 4.5)/\tanh(1.5)$$
$$f(r < 5) = f(5) \qquad f(r > 10) = 0 \tag{S.2}$$

TABLE S.2: Optimized parameters of bridge function $B_u(r)$ for amino acid segments.

amino acid segment	σ_i(Å)	ϵ_i	ξ_i
Val	4.4289	1.5348	0.0678
Leu	4.6952	1.6724	0.0771
Ile	4.7650	1.7288	0.0813
Cys	4.2850	1.8360	0.0901
Ala	5.0619	1.9736	0.1042
Met	4.7696	1.9988	0.1074
Ser	4.1381	2.1468	0.1243
Thr	4.4190	2.1672	0.1257
Gln	4.6996	2.7900	0.1684
Asn	4.4061	2.8192	0.1705
Glu	4.3501	3.1576	0.1957
Asp	4.3549	3.2448	0.2025
Asn	4.4061	2.8193	0.1705
Ly2	4.3383	2.9384	0.1792
Glu	4.3501	3.1576	0.1957
Ar1	4.3777	1.2415	0.0510
Ar2	4.9237	2.9647	0.1812
Gbb	4.0542	1.9518	0.1017
Hi1	4.0437	1.0570	0.0418
Hi2	4.1159	2.2062	0.1282
Ber	3.9400	0.9378	0.0363
Xyr	4.1350	1.5300	0.0675
Pro	4.4289	1.5347	0.0678
Tr1	4.3202	1.4173	0.0607
Tr2	4.2597	1.3094	0.0546
Tr3	4.5598	1.9205	0.0983
Phr	4.0331	2.0584	0.1161
Gbm	3.9639	1.6002	0.0721

The KH expression for the free energy is expressed as

$$G_{KH} = 0.5k_bT\rho_0 \int [\Theta(-h(\mathbf{r}))h^2(\mathbf{r}) - 2c(\mathbf{r}) - h(\mathbf{r})c(\mathbf{r})]d\mathbf{r} \tag{S.3}$$

In the case of nonzero bridge, the general expression for the free energy involves integration over the parameter λ of thermodynamic integration. The bridge contribution can be written as

$$G_B = k_bT\rho_0 \int [B(\mathbf{r}) + \int_0^1 d\lambda h(\lambda,\mathbf{r}) \frac{dB(\lambda,\mathbf{r})}{d\lambda}]d\mathbf{r} \tag{S.4}$$

Assuming the linear dependence between $h(\lambda,\mathbf{r})$ and λ, i.e. $h(\lambda,\mathbf{r}) = \lambda h(\mathbf{r})$, the similar relations for indirect correlation function and bridge functions $B_u(r)$, we can integrate analytically and finally obtain

$$\int\int_0^1 d\lambda h(\lambda,\mathbf{r}) \frac{dB(\lambda,\mathbf{r})}{d\lambda} d\mathbf{r} \equiv \int h(\mathbf{r})[B_a(\gamma(\mathbf{r})) + 0.5B_u((\mathbf{r})) - f_e(\gamma(\mathbf{r}))]d\mathbf{r}$$
$$f_e(\gamma) = \Theta(-\gamma)[\frac{A\gamma}{2C} - \frac{A}{C^2}(1 - \frac{\ln(1+C\gamma)}{C\gamma})] \tag{S.5}$$

where $\Theta(\gamma)$ is the Heaviside function.

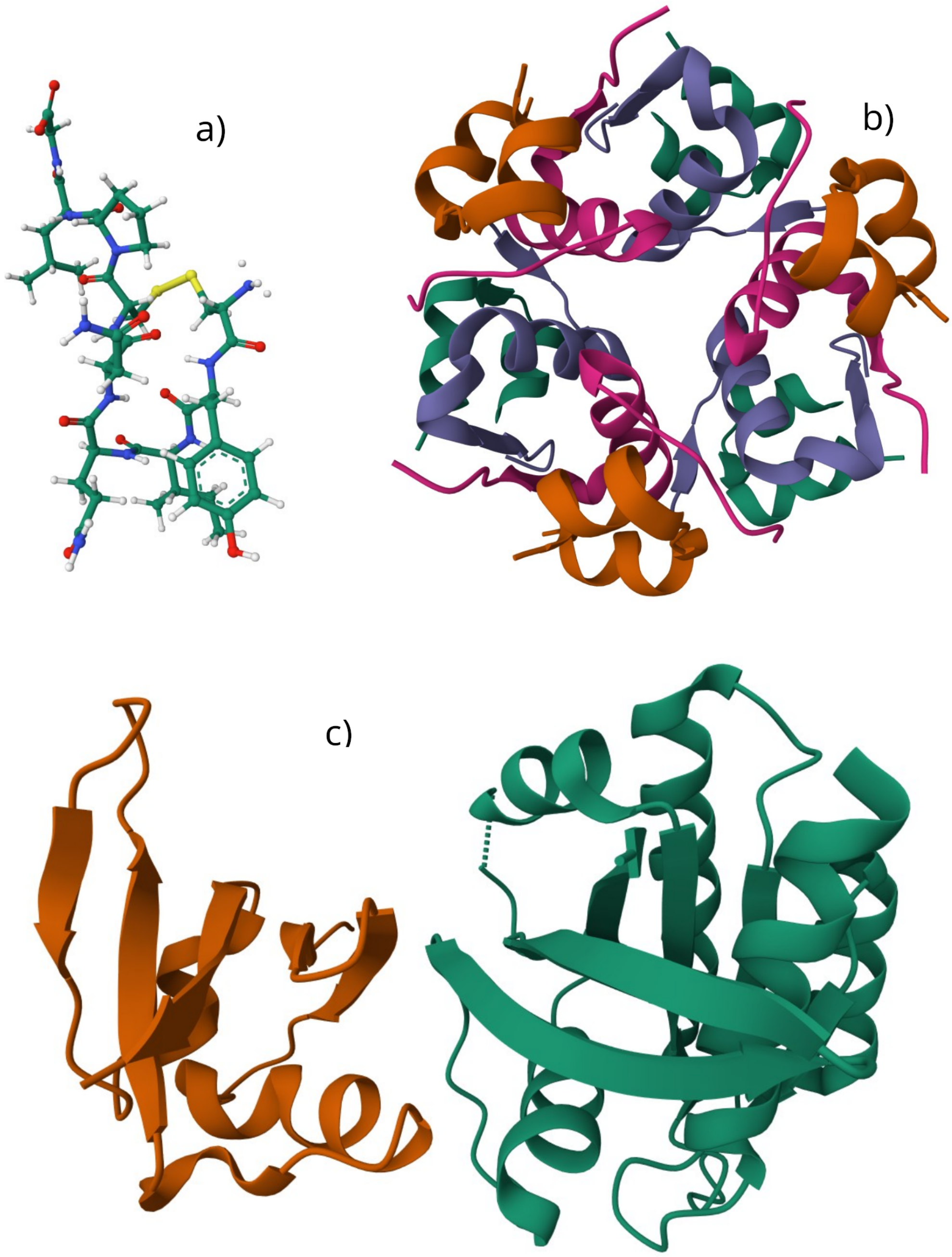

FIG. S.3: Molecular structure of oxytocin (a), insulin (b), and Ras-Rasf dimer (c) derived from the pdb database.
